# Trends and risk factors of hypertension in US Children and Adolescents, 1999–2023

**DOI:** 10.1038/s41371-025-01102-9

**Published:** 2025-12-05

**Authors:** Daniel Ouyang, Wentao Cao, Yun Shen, Liwei Chen, Gang Hu

**Affiliations:** 1 Thomas S. Wootton High School, Rockville, MD USA; 2 Pennington Biomedical Research Center, Baton Rouge, LA USA; 3 Department of Epidemiology, Fielding School of Public Health, University of California Los Angeles, Los Angeles, CA USA

**Keywords:** Hypertension, Risk factors

## Abstract

We aimed to assess trends in elevated blood pressure (EBP) and hypertension among US children and adolescents before and after the COVID-19 pandemic using data from 25,916 participants aged 8-19 years in NHANES 1999–2023. Survey-weighted multinomial logistic regression was used to examine associations of sociodemographic, nutritional and other factors with EBP and hypertension overall and across subgroups during the pre-pandemic cycles (2015-2020) and post-pandemic cycles (2021–2023). Among children (n = 10,616), EBP prevalence decreased from 4.3% in 1999–2002 to 3.5% in 2021–2023 (P = 0.36), and hypertension declined from 3.3% to 2.3% (P = 0.025). Among adolescents (n = 15,300), EBP declined from 10.0% to 9.4% (P = 0.46), and hypertension prevalence fell from 8.3% to 5.1% (P < 0.001). From 2015–2023, obesity was strongly associated with both EBP and hypertension in children (odds ratio [OR] 1.78, 95% CI 1.02–3.10) and adolescents (OR 1.89, 95% CI 1.30-2.74). In children, higher dietary fat intake was associated with greater odds of EBP, and higher sodium intake with greater odds of hypertension. In adolescents, older age, male sex and non-Hispanic Black race were additional risk factors. Comparing pre-pandemic (2015-2020) with post-pandemic (2021–2023) cycles, EBP prevalence in adolescents decreased (11.6% vs 9.42%, P = 0.46) and hypertension prevalence in children changed modestly (2.53% vs 2.26%, P = 0.025). Despite concerns about pandemic-related increases in obesity, pediatric EBP and hypertension prevalence remained stable or declined from 2015 to 2023, with adiposity remaining the dominant modifiable correlate.

## Introduction

High blood pressure in children is a prominent risk factor for cardiovascular diseases including hypertension in adulthood [1]. Childhood hypertension is linked with target organ damage, left ventricular hypertrophy, and subclinical atherosclerosis [1–4]. The rate of elevated blood pressure (EBP) and hypertension among aged 30–79 years old has doubled from 1990–2019, affecting more than 1 billion people worldwide [5, 6]. Research has shown that the prevalence of EBP and hypertension in adolescent has decreased from 1999 to 2018 and their risks are associated with a higher body mass index (BMI) and lower physical activity [1, 7].

The coronavirus pandemic, starting in 2019 (COVID-19) largely impacted the physical activity, diet, stress, and lifestyle of adolescents [8–12]. This includes declines in physical activity and quality nutrition, which was followed by a spike in obesity rates among adolescents [11]. It was also reported that obese children experienced an even higher increase in BMI during the pandemic [8]. Specifically, prevalence of obesity rates in US children aged 2 to 19 years grew from 16.9% in 2011–2012 to 19.7% in 2017-2020 and then to 21.1% in 2021-2023 periods [11]. Previously, adequate data to compare prevalence of hypertension or high blood pressure before and after COVID-19 in US children and adolescents were not available. The present study aimed to assess the trends of EBP and hypertension among US adolescents before and after COVID using recent release of data from the US National Health and Nutrition Examination Survey (NHANES) and to examine the major risk factors associated with EBP and hypertension.

## Methods

### Data source

This study utilized data from the NHANES, which collects individual-level demographic, health, and nutrition information through personal interviews and physical examinations [13]. The data are used to estimate the prevalence of various diseases and conditions and to support health policy planning.

### Study population

**Fig. 1:**
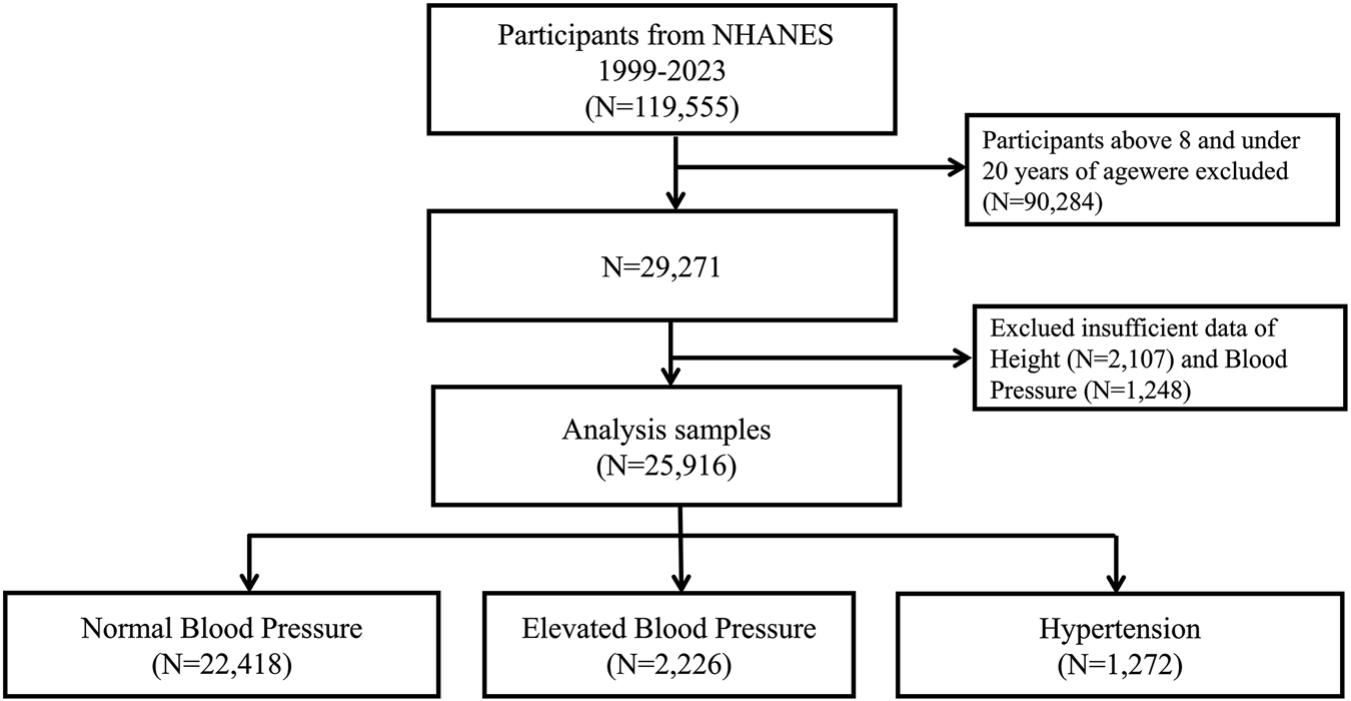
Flow chart of the study.

The study sample included 25,916 participants aged 8 to 19 years who completed demographic and dietary interviews and underwent physical examinations as part of NHANES cycles from 1999 to 2023 (see Figure 1). The response rate was 76.0% in 1999–2000 and a sharp decline of 25.7% in 2021–2023. Examinations included standardized assessments of height, weight, and systolic and diastolic blood pressure. Participants with missing data on blood pressure (systolic or diastolic) or weight/height were excluded from the analysis. The Pennington Biomedical Research Center Institutional Review Board deemed this study as non-human participant research.

### Sociodemographic, health and dietary characteristics

The parent-reported sociodemographic information collected during the interview included the child’s age, gender, race, and poverty-to-income ratio. Weight and height are measured with specialized equipment in a mobile examination center. Body mass index (BMI) was calculated by dividing weight in kilograms by the square of height in meters. BMI categorized weight status into normal, overweight and obesity based on Z-scores of BMI calculated using CDC BMI-for Age and Sex growth charts data file [14]. According to Clinical Practice Guideline [15], normal weight was defined as BMI < 85 percentiles (Z score of BMI < 1.035); overweight was defined as BMI ≥ 85 percentiles and <95 percentiles (Z score of BMI ≥ 1.035 and <1.645); and obesity was defined as BMI ≥ 95 percentiles (Z score of BMI ≥ 1.645). The dietary characteristics included daily energy, fat, saturated fatty acids, dietary fiber, sodium and potassium. The dietary intake data are used to estimate the types and amounts of foods and beverages consumed during the 24-hour period prior to the interview. Intakes of energy, nutrients, and other food components are then calculated using the Food and Nutrient Database for Dietary Studies (FNDDS). Interviews used age‑specific respondent protocols. For participants under 6 years, a proxy completed the interview. For children 6–8 years, the proxy was the primary respondent with the child present to assist. For children 9–11 years, the child was the primary respondent with assistance from a proxy familiar with the child’s intake. Participants ≥12 years completed the interview themselves. Daily intakes of sodium and potassium for each individual were estimated by summing the amounts consumed from all reported foods and beverages for 24-hour period. We analyzed total energy, total fat (as % of energy), saturated fat (as % of energy), dietary fiber (g/day), sodium (mg/day), and potassium (mg/day) [16].

#### Blood pressure measurement and definitions

In all NHANES cycles, trained staff measured upper arm circumference prior to blood pressure measurements and selected an appropriately sized cuff per NHANES protocol (cuff size 2 for those with upper arm circumferences 17–21.9 cm; size 3 for 22–31.9 cm; size 4 for 32–41.9 cm and size 5 for 42–50 cm). Blood pressure was traditionally measured using a mercury sphygmomanometer. Following the 2017–2018 survey cycle, NHANES replaced the mercury sphygmomanometer with oscillometric devices for collecting auscultatory BP measurements. After the participant rests quietly in a seated position for five minutes, three consecutive BP readings are taken. The average of these three measurements was used to determine systolic blood pressure (SBP) and diastolic blood pressure (DBP). According to the 2017 American Academic of Pediatrics Clinical Practice Guideline, the BP status categorized into normal BP, elevated BP, and hypertension [17]. For children aged 8 to 12 years, SBP and DBP percentiles were determined using age-, sex-, and height-specific reference tables [18]. Normal BP was defined as SBP and DBP below the 90^th^ percentile. Elevated BP was defined as SBP and/or DBP between the 90^th^ and 95^th^ percentiles. Hypertension was defined as SBP and/or DBP at or above the 95^th^ percentile, or an SBP of at least 130 mm Hg and/or a DBP of at least 80 mm Hg. For adolescents aged 13 to 19 years, normal BP was defined as SBP less than 120 mm Hg and DBP less than 80 mm Hg; elevated BP as SBP 120 to 129 mm Hg with DBP less than 80 mm Hg; and hypertension as SBP at least 130 mm Hg and/or DBP at least 80 mm Hg.

##### Statistical analysis

All analyses were conducted separately for children aged 8 to 12 years and adolescents aged 13 to 17 years using data from 6 combined NHANES cycles spanning 1999 to 2023 (1999–2002, 2003–2006, 2007–2010, 2011–2014, 2015–2020, 2021–2023). The complex, multistage sampling design of NHANES was accounted for by applying appropriate survey weights, strata, and primary sampling units, in accordance with National Center for Health Statistics guidelines. These weights ensured nationally representative estimates for U.S. children and adolescents.

Depending on variable type, either a design-based one-way ANOVA or a Rao–Scott adjusted chi-square test was used to evaluate trends across the NHANES cycles. Estimates are presented for the overall population and stratified by predefined subgroups, including sex, race, BMI, DBP, SBP, poverty-income ratio (PIR), and selected dietary factors. The Rao–Scott adjusted chi-square test of independence was also used to evaluate differences in prevalence of EBP and hypertension across the NHANES cycles.

The survey-weighted multinomial logistic regression was conducted to examine associations of sociodemographic, health and nutrition factors with odd ratios of EBP and hypertension across subgroup listed in Table 3 during pre‑pandemic cycle (2015–2020), the post‑pandemic cycle (2021–2023) and combined cycle. All covariate listed in Table 3 are included in the multinomial logistic regression. Data analysis was conducted using R, version 4.4.3. Statistical significance was p < 0.05.

## Results

In the 1999–2002 cycle, among US children aged 8 to 12 years (mean age of 10.1 years), 51.7% were boys and 48.3% were girls; 59.4% were non-Hispanic White, 19.8% were Hispanic, 16.1% were non-Hispanic Black, and 4.7% were of other ethnicities. In the 2021–2023 cycle, among US children aged 8 to 12 years (mean age of 10.0 years), 52.6% were boys, 47.4% were girls; 46.1% were non-Hispanic White, 24.6% were Hispanic, 12.9% were non-Hispanic Black, and 16.4% were of other ethnicities (Table 1).

**Table 1:** Characteristics of US children and adolescents from 1999 to 2023.

	NHANES Cycle						P value
	1999–2002	2003–2006	2007–2010	2011–2014	2015–2020	2021–2023	
**Children aged 8**–**12 years old**							
No. of participants	2021	1880	1853	1924	2217	721	
Age, years, mean (SD)	10.1 (1.44)	10.0 (1.40)	10.0 (1.42)	10.1 (1.43)	10.0 (1.41)	10.0 (1.45)	0.90
Sex, No (%)							0.70
Boys	1012 (51.7)	918 (51.8)	928 (50.6)	975 (50.5)	1099 (49.3)	366 (52.6)	
Girls	1009 (48.3)	962 (48.2)	925 (49.4)	949 (49.5)	1118 (50.7)	355 (47.4)	
Race/ethnicity, No (%)							0.005
Non-Hispanic white	520 (59.4)	478 (58.7)	574 (57.2)	501 (54.6)	642 (50.6)	287 (46.1)	
Hispanic	787 (19.8)	663 (17.6)	758 (21.6)	615 (23.2)	672 (25.4)	203 (24.6)	
Non-Hispanic black	648 (16.1)	627 (15.2)	414 (13.6)	518 (13.5)	544 (13.3)	117 (12.9)	
Other	66 (4.70)	112 (8.50)	107 (7.60)	290 (8.70)	359 (10.7)	114 (16.4)	
Body mass index, No (%)							0.003
Normal	1267 (66.6)	1128 (63.5)	1103 (61.6)	1098 (59.5)	1256 (60.3)	393 (55.9)	
Overweight	329 (16.2)	325 (17.5)	321 (17.4)	352 (17.7)	401 (17.5)	115 (16.3)	
Obesity	425 (17.3)	427 (19.1)	429 (21.0)	474 (22.8)	560 (22.3)	213 (27.8)	
Blood pressure, mean (SD)							
Systolic, mmHg	103 (8.95)	103 (9.69)	102 (9.43)	102 (8.93)	102 (8.92)	102 (8.57)	0.012
Diastolic, mmHg	56.8 (12.5)	53.3 (13.3)	52.2 (14.6)	51.0 (15.7)	57.0 (10.9)	60.5 (7.14)	<0.001
Poverty-to-income ratio, No (%)							0.200
<1.30	836 (34.9)	727 (28.7)	774 (32.7)	878 (36.5)	783 (28.0)	219 (26.8)	
1.30–3.49	640 (37.1)	690 (40.1)	603 (36.0)	558 (34.3)	795 (40.0)	252 (40.2)	
≥3.5	352 (27.9)	387 (31.2)	342 (31.3)	379 (29.2)	424 (32.1)	167 (33.0)	
Dietary factors, mean (SD)							
Energy, kcal/d	2060 (79)	2101 (755)	1963 (72)	1994 (765)	1998 (792)	1937 (710)	<0.001
Fat, % energy	32.4 (7.72)	33.5 (7.32)	32.6 (7.21)	33.5 (7.23)	35.1 (7.12)	36.0 (7.54)	<0.001
Saturated fat, % energy	11.6 (3.50)	11.8 (3.39)	11.4 (3.36)	11.7 (3.47)	12.2 (3.60)	12.4 (3.86)	<0.001
Fiber, g/d	12.6 (7.22)	13.3 (7.00)	13.4 (7.03)	14.6 (7.54)	15.1 (8.71)	15.2 (8.25)	<0.001
Sodium, mg/d	3201 (1557)	3255 (1441)	3106 (1441)	3140 (1398)	3124 (1432)	2864 (1219)	0.001
Potassium, mg/d	2232 (1097)	2193 (982)	2106 (898)	2200 (935)	211 (955)	2021 (832)	0.001
Na/K ratio	1.61 (0.78)	1.63 (0.73)	1.58 (0.63)	1.53 (0.62)	1.60 (0.69)	1.52 (0.61)	0.035
**Children aged 13–19 years old**							
No. of participants	3910	3735	2060	2167	2501	927	
Age, years, mean (SD)	15.9 (1.98)	15.9 (1.98)	15.9 (1.93)	15.9 (1.97)	15.8 (1.94)	16.0(2.00)	0.46
Sex, No (%)							0.76
Boys	1964 (51.1)	1868 (49.8)	1118 (52.3)	1091 (50.3)	1291 (51.6)	461 (51.0)	
Girls	1946 (48.9)	1867 (50.2)	942 (47.7)	1076 (49.7)	1210 (48.4)	466 (49.0)	
Race/ethnicity, No (%)							<0.001
Non-Hispanic white	968 (59.3)	1015 (64.4)	668 (60.1)	514 (54.6)	765 (53.5)	361 (45.5)	
Hispanic	1654 (18.1)	1280 (15.6)	776 (18.8)	673 (21.6)	689 (22.8)	297 (25.7)	
Non-Hispanic black	1119 (13.5)	1293 (14.7)	504 (14.8)	596 (14.9)	607 (13.3)	120 (11.1)	
Other	169 (9.10)	147 (5.30)	112 (6.30)	384 (9.00)	440 (10.4)	149 (17.7)	
Body mass index, No (%)							<0.001
Normal	2530 (69.3)	2349 (65.9)	1294 (66.1)	1332 (62.5)	1398 (57.6)	536 (60.0)	
Overweight	624 (14.5)	634 (16.6)	334 (15.2)	371 (16.1)	482 (19.5)	154 (16.5)	
Obesity	756 (16.3)	752 (17.6)	432 (18.7)	464 (21.5)	621 (23.0)	237 (23.5)	
Blood pressure, mean (SD)							
Systolic, mmHg	110 (10.3)	110 (10.4)	110 (10.3)	109 (9.91)	110(9.77)	108 (10.3)	0.027
Diastolic, mmHg	63.2(11.5)	60.4 (11.7)	60.4 (11.7)	60.5 (11.9)	62.9 (9.15)	64.7 (8.30)	<0.001
Poverty-to-income ratio, No (%)							0.37
<1.30	1521 (33.2)	1492 (29.3)	789 (30.4)	895 (33.6)	900 (28.1)	277 (28.3)	
1.30–3.49	1266 (35.3)	1284 (36.8)	675 (34.4)	668 (37.3)	846 (37.8)	331 (41.9)	
≥3.5	736 (31.5)	766 (33.9)	408 (35.2)	411 (29.1)	502 (34.1)	210 (29.8)	
Dietary factors, mean (SD)							
Energy, kcal/d	2377 (1161)	2372 (1157)	2230 (1091)	2145 (993)	2112 (1031)	2030 (985)	<0.001
Fat, % energy	31.9 (8.43)	33.3 (8.15)	32.8 (8.37)	33.1 (8.10)	35.9 (8.33)	36.2 (8.93)	0.001
Saturated fat, % energy	11.1 (3.64)	11.5 (3.53)	11.2 (3.58)	11.1 (3.70)	12.2 (4.05)	12.1 (4.31)	<0.001
Fiber, g/d	13.8 (9.51)	13.9 (8.87)	14.3 (9.17)	14.7 (8.76)	14.6 (8.73)	15.3 (9.92)	0.016
Sodium, mg/d	3555 (2065)	3645 (2045)	3637 (2049)	3550 (1829)	3458 (1854)	3120 (1653)	<0.001
Potassium, mg/d	2495 (1451)	2438 (1389)	2337 (1288)	2334 (1186)	2203 (1234)	2172 (1322)	<0.001
Na/K ratio	1.60 (0.78)	1.66 (0.75)	1.70 (0.78)	1.65 (0.71)	1.75 (0.86)	1.62 (0.73)	<0.001

Data were obtained from the National Health and Nutrition Examination Survey. For categorical variables, results are presented as unweighted frequencies (N) and weighted percentages (%); for continuous variables, weighted means and standard deviations are reported.

Among adolescents aged 13 to 19 years in the 1999–2002 cycle (mean age of 15.9 years), 51.1% were boys and 48.9% were girls; 59.3% were non-Hispanic White, 18.1% were Hispanic, 13.5% were non-Hispanic Black, and 9.10% were of other ethnicities. Among adolescents aged 13 to 19 years in the 2021–2023 cycle (mean age of 16.0 years), 51.0% were boys and 49.0% were girls; 45.5% were non-Hispanic White, 25.7% were Hispanic, 11.1% were non-Hispanic Black, and 17.7% were other of other ethnicities (Table 1) Fig. 1.

### Trends in prevalence of elevated blood pressure and hypertension

**Fig. 2:**
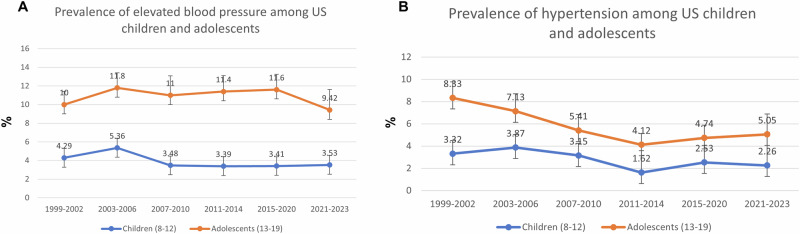
Prevalence of elevated blood pressure and hypertenion among US children and adolescents. **A** Prevalence of elevated blood pressure among US children and adolescents; **B** Prevalence of hypertension among US children and adolescents.

The prevalence of EBP for US children aged 8 to 12 years old increased from 4.29% (2.81%–5.77%) in 1999–2002 to 5.36% (3.53%–7.18%) in 2003–2006 and then decreased to 3.53% (1.40%–5.65%) in 2021–2023 (P = 0.36) (Table 2). In 2007–2010, 2011–2014, 2015–2020, and 2021–2023 the prevalence of EBP in children remained stable. The prevalence of EBP for US adolescents aged 13 to 19 years old increased from 10.0% (8.59%–11.4%) in 1999–2002 to 11.8% (10.2%–13.4%) in 2003–2006 and then decreased to 9.42% (7.22%–11.6%) in 2021–2023 (P = 0.46). In 2003–2006, 2007–2010, 2011–2014, and 2015–2020, the prevalence of EBP in adolescents remained stable (Fig. 2A).

**Table 2:** Prevalence of elevated blood pressure and hypertension among US children and adolescents from 1999 to 2023.

	NHANES Cycle						P value
	1999–2002	2003–2006	2007–2010	2011–2014	2015–2020	2021–2023	
**Children aged 8**–**12 years old**							
Elevated blood pressure							
Total	4.29 (2.81–5.77)	5.36 (3.53–7.18)	3.48 (2.08–4.88)	3.39 (2.25–4.54)	3.41 (2.23–4.59)	3.53 (1.40–5.65)	0.36
Boys	4.46 (2.44–6.49)	5.63 (2.90–8.37)	3.53 (1.92–5.14)	2.98 (1.55–4.41)	3.07 (1.95–4.19)	2.88 (0.61–5.14)	0.24
Girls	4.11 (2.41–5.81)	5.06 (3.28–6.84)	3.43 (1.90–4.96)	3.82 (2.05–5.58)	3.73 (1.78–5.69)	4.24 (0.78–7.71)	0.89
Race/ethnicity							
Non-Hispanic white	3.89 (1.93–5.84)	4.99 (2.48–7.51)	2.82 (1.12–4.53)	3.43 (1.47–5.39)	3.11 (1.59–4.63)	3.76 (0.80–6.72)	0.70
Hispanic	4.15 (2.20–6.09)	6.57 (3.76–9.38)	4.21 (1.82–6.60)	2.84 (1.31–4.37)	3.69 (2.32–5.05)	1.85 (0.60–3.09)	0.06
Non-Hispanic black	5.91 (3.60–8.22)	4.99 (3.42–6.56)	3.71 (1.87–5.54)	3.33 (1.63–5.03)	2.23 (1.20–3.27)	2.34 (0.00–5.10)	0.05
Other	4.47 (0.00–9.97)	5.99 (0.90–11.09)	5.96 (0.34–11.58)	4.71 (2.88–6.53)	5.58 (0.00–11.6)	6.30 (0.00–13.1)	0.99
Hypertension							
Total	3.32 (2.15–4.49)	3.87 (2.71–5.02)	3.15 (2.06–4.23)	1.62 (0.94–2.30)	2.53 (1.66–3.39)	2.26 (1.26–3.26)	0.025
Boys	3.58 (2.07–5.09)	3.56 (2.00–5.13)	2.15 (1.06–3.25)	1.81 (0.78–2.83)	2.13 (1.24–3.01)	2.49 (1.31–3.68)	0.14
Girls	3.03 (1.24–4.82)	4.19 (2.27–6.11)	4.17 (2.18–6.15)	1.43 (0.54–2.32)	2.91 (1.59–4.23)	2.00 (0.32–3.69)	0.11
Race/ethnicity							
Non-Hispanic white	3.47 (1.72–5.21)	4.10 (2.27–5.92)	3.36 (1.60–5.11)	1.11 (0.04–2.17)	2.18 (1.01–3.36)	1.84 (0.54–3.14)	0.06
Hispanic	3.46 (1.71–5.20)	3.07 (1.08–5.05)	2.61 (1.14–4.07)	2.19 (0.99–3.38)	2.74 (1.80–3.68)	1.85 (0.29–3.42)	0.77
Non-Hispanic black	2.22 (0.60–3.84)	4.22 (2.65–5.78)	3.66 (2.04–5.28)	1.80 (0.15–3.46)	3.21 (1.38–5.05)	4.95 (0.00–10.2)	0.40
Other	4.60 (0.00–12.5)	3.31 (0.00–6.89)	2.17 (0.00–5.77)	3.07 (0.16–5.97)	2.78 (0.00–6.60)	1.94 (0.00–4.14)	0.96
**Adolecents aged 13**–**19 years old**							
Elevated blood pressure							
Total	10.0 (8.59–11.4)	11.8 (10.2–13.4)	11.0 (8.92–13.0)	11.4 (9.70–13.0)	11.6 (9.95–13.3)	9.42 (7.22–11.6)	0.46
Boys	14.7 (12.2–17.2)	16.4 (14.2–18.6)	16.2 (13.0–19.4)	16.7 (13.9–19.6)	17.6 (15.0–20.3)	15.3 (12.1–18.4)	0.70
Girls	5.12 (3.90–6.35)	7.27 (5.48–9.06)	5.21 (3.66–6.76)	5.93 (4.11–7.76)	5.27 (3.69–6.85)	3.34 (1.33–5.36)	0.13
Race/ethnicity							
Non-Hispanic white	9.85 (7.66–12.0)	11.1 (9.19–13.1)	9.48 (7.03–11.9)	11.0 (8.37–13.6)	12.5 (9.70–15.2)	8.16 (5.13–11.2)	0.32
Hispanic	9.51 (7.22–11.8)	11.8 (9.52–14.1)	11.4 (8.29–14.4)	12.0 (8.65–15.4)	7.97 (5.76–10.2)	9.61 (4.89–14.3)	0.26
Non-Hispanic black	13.1 (10.9–15.3)	16.0 (13.7–18.3)	15.3 (11.9–18.7)	14.0 (11.5–16.4)	16.2 (13.5–18.9)	9.69 (2.55–16.8)	0.31
Other	7.54 (3.66–11.4)	8.59 (2.32–14.9)	13.7 (5.60–21.7)	7.80 (4.40–11.2)	9.63 (5.99–13.3)	12.2 (6.82–17.6)	0.49
Hypertension							
Total	8.33 (6.90–9.76)	7.13 (5.56–8.70)	5.41 (3.98–6.85)	4.12 (3.10–5.15)	4.74 (3.60–5.88)	5.05 (3.21–6.90)	<0.001
Boys	11.7 (10.2–13.1)	10.3 (7.89–12.7)	7.79 (5.69–9.90)	6.48 (4.50–8.46)	6.69 (5.13–8.25)	6.03 (3.57–8.49)	<0.001
Girls	4.83 (2.67–6.99)	4.00 (2.76–5.25)	2.80 (1.64–3.95)	1.74 (0.82–2.67)	2.65 (1.01–4.30)	4.04 (1.69–6.39)	0.075
Race/ethnicity							
Non-Hispanic white	8.09 (6.52–9.66)	7.77 (5.36–10.2)	5.46 (3.30–7.63)	2.91 (1.26–4.56)	4.62 (2.45–6.78)	4.84 (2.57–7.11)	0.006
Hispanic	5.70 (4.28–7.13)	4.32 (2.93–5.72)	3.79 (2.66–4.93)	4.44 (2.73–6.15)	4.85 (3.02–6.68)	3.93 (1.63–6.24)	0.67
Non-Hispanic black	10.7 (8.70–12.7)	8.25 (6.10–10.4)	9.14 (5.60–12.7)	7.16 (4.40–9.93)	6.37 (4.05–8.68)	7.50 (3.83–11.2)	0.27
Other	11.6 (3.51–19.7)	4.51 (0.71–8.30)	0.96 (0.00–2.35)	5.73 (2.00–9.46)	3.02 (1.79–4.24)	5.68 (0.45–10.9)	0.015

Prevalences were % (95% confidence intervals); all estimates were weighed.

The prevalence of hypertension for US children aged 8 to 12 years old increased from 3.32% (2.15%–4.49%) in 1999–2002 to 3.87% (2.71%–5.02%) in 2003–2006 and then decreased to 3.15% (2.06%–4.23%) in 2007–2010 to 1.62% (0.94%–2.30%) in 2011–2014 and then increased to 2.53% (1.66%–3.39%) in 2015–2020 and then decreased to 2.26% (1.26%–3.26%) in 2021–2023 (P = 0.025) (Table 2). The prevalence of hypertension for US adolescents aged 13 to 19 years old decreased from 8.33% (6.90%–9.76%) in 1999–2002 to 7.13% (5.56%–8.70%) in 2003–2006 to 4.12% (3.10%–5.15%) in 2011–2014 and then increased to 4.74% (3.60%–5.88%) in 2015–2020 to 5.05% (3.21%–6.90%) in 2021–2023 (P ≤ 0.001) (Fig. 2B).

### Factors associated with elevated blood pressure and hypertension in US children and adolescents from 2015–2023

Among US children aged 8 to 12 years old, those who were obese had higher odds of having EBP than those with normal weight (OR, 1.78; 95% Cl, 1.02–3.10) (Table 3). Children whose poverty-to-income ratio was greater or equal to 3.5 had lower odds of having hypertension (OR, 0.15; 95% Cl, 0.05–0.44) compared with those with poverty-to-income ratio <1.3 (Table 3). Furthermore, each 1% increase in fat as a percentage of total energy intake was associated with higher odds of EBP (OR, 1.05; 95% CI, 1.01–1.09), and each 100 mg increase in daily dietary sodium intake was also associated with an increased risk of EBP (OR, 1.03; 95% CI, 1.00–1.06). No other factor such as age, race, and other dietary factors was significant. Among US adolescents aged 13–19 years old, every additional year of age was associated with higher odds of EBP (OR, 1.26; 1.14–1.40) and hypertension (OR, 1.29; 1.08–1.53) (Table 3). Additionally, girls had lower odds of having EBP and hypertension than boys (OR, 0.25; 95% Cl, 0.17–0.35 and OR, 0.31; 95% Cl, 0.15–0.64, respectively). Adolescents who were non-Hispanic Black had higher odds of having EBP than those who were non-Hispanic White (OR, 1.43; 95% Cl, 1.02–2.02). Adolescents who were overweight and obese had higher odds of EBP compared to those who were normal weight (OR, 1.82; 95% Cl, 1.19–2.79 and OR, 1.82; 95% Cl, 1.89; 95% Cl, 1.30–2.74, respectively). Adolescents who were obese had higher odds of hypertension compared to those who were normal weight (OR, 3.69; 95% Cl, 2.09–6.52). No other factor such as poverty-to-income ratio, NHANES cycle (before and post COVID), or dietary factors were significant.

**Table 3:** Adjusted odds ratios (OR) and 95% confidence intervals (CI) for elevated blood pressure and hypertension among US children and adolescents.

	NHANES 2015–2023		NHANES 2015–2020		NHANES 2021–2023	
	EBP	Hypertension	EBP	Hypertension	EBP	Hypertension
**Children aged 8**–**12 years old**						
Age	0.84 (0.64–1.10)	0.91 (0.69–1.20)	0.87 (0.63–1.22)	0.98 (0.71–1.35)	0.79 (0.57–1.10)	0.67 (0.44–1.04)
Sex						
Boys	1.00	1.00	1.00	1.00	1.00	1.00
Girls	1.58 (0.81–3.10)	1.11 (0.63–1.95)	1.24 (0.59–2.61)	1.28 (0.70–2.35)	3.73 (0.66–21.0)	0.73 (0.20–2.62)
NHANES Cycle						
NHANES 2015–2020	1.00	1.00	NA	NA	NA	NA
NHANES 2021–2023	0.95 (0.42–2.16)	0.99 (0.50–1.95)	NA	NA	NA	NA
Race/ethnicity						
Non-Hispanic white	1.00	1.00	1.00	1.00	1.00	1.00
Hispanic	0.91 (0.49–1.70)	0.77 (0.42–1.41)	1.29 (0.66–2.51)	0.71 (0.35–1.42)	0.17 (0.02–1.71)	0.76 (0.23–2.51)
Non-Hispanic black	0.59 (0.28–1.24)	0.70 (0.31–1.61)	0.60 (0.28–1.29)	0.54 (0.25–1.18)	0.71 (0.14–3.60)	1.20 (0.22–6.58)
Other	2.07 (0.88–4.89)	1.29 (0.38–4.40)	1.94 (0.63–5.94)	1.37 (0.27–6.99)	2.11 (0.53–8.31)	1.42 (0.31–6.57)
Body mass index						
Normal	1.00	1.00	1.00	1.00	1.00	1.00
Overweight	1.73 (0.81–3.68)	0.63 (0.22–1.84)	1.66 (0.69–3.95)	0.72 (0.21–2.47)	2.15 (0.55–8.43)	0.35 (0.04–2.96)
Obesity	1.78 (1.02–3.10)	2.32 (0.94–5.72)	1.54 (0.82–2.89)	2.22 (0.75–6.59)	3.21 (1.63–6.33)	3.06 (0.74–12.6)
Poverty-to-income ratio						
<1.30	1.00	1.00	1.00	1.00	1.00	1.00
1.30–3.49	0.72 (0.38–1.36)	0.77 (0.44–1.34)	0.79 (0.41–1.54)	0.71 (0.36–1.39)	0.48 (0.14–1.66)	0.82 (0.30–2.24)
≥3.5	0.87 (0.36–2.10)	0.15 (0.05–0.44)	1.12 (0.46–2.73)	0.06 (0.02–0.21)	0.49 (0.08–3.08)	0.37 (0.07–1.88)
Dietary factors						
Energy, 100 kcal	0.92 (0.85–1.00)	0.88 (0.77–1.00)	0.94 (0.87–1.02)	0.87 (0.74–1.01)	0.82 (0.65–1.02)	0.93 (0.78–1.10)
Fat, % energy	1.05 (1.01–1.09)	1.03 (0.96–1.09)	1.04 (0.99–1.09)	1.04 (0.96–1.13)	1.08 (0.98–1.19)	0.96 (0.90–1.04)
Saturated fat, % energy	0.99 (0.89–1.11)	1.05 (0.97–1.14)	1.00 (0.88–1.14)	1.01 (0.91–1.12)	0.95 (0.73–1.24)	1.14 (1.00–1.30)
Fiber, g/d	0.98 (0.91–1.07)	1.02 (0.97–1.08)	0.98 (0.89–1.07)	1.03 (0.96–1.09)	1.01 (0.89–1.14)	1.00 (0.89–1.12)
Sodium, 100 mg/d	1.03 (1.00–1.06)	1.03 (1.00–1.07)	1.02 (1.00–1.05)	1.03 (0.98–1.08)	1.07 (0.96–1.19)	1.05 (1.01–1.10)
Potassium, 100 mg/d	1.04 (0.97–1.12)	1.01 (0.96–1.07)	1.05 (0.98–1.12)	1.04 (0.97–1.11)	1.03 (0.90–1.19)	0.94 (0.87–1.02)
**Adolescents aged 13**–**19 years old**						
Age	1.26 (1.14–1.40)	1.29 (1.08–1.53)	1.25 (1.11–1.41)	1.22 (0.99–1.50)	1.36 (1.11–1.67)	1.72 (1.36–2.17)
Sex						
Boys	1.00	1.00	1.00	1.00	1.00	1.00
Girls	0.25 (0.17–0.35)	0.31 (0.15–0.64)	0.26 (0.17–0.39)	0.31 (0.13–0.75)	0.17 (0.09–0.33)	0.33 (0.15–0.75)
NHANES Cycle						
NHANES 2015–2020	1.00	1.00	NA	NA	NA	NA
NHANES 2021–2023	0.69 (0.44–1.10)	0.87 (0.51–1.47)	NA	NA	NA	NA
Race/ethnicity						
Non-Hispanic white	1.00	1.00	1.00	1.00	1.00	1.00
Hispanic	0.67 (0.45–0.98)	0.65 (0.36–1.15)	0.60 (0.40–0.91)	0.63 (0.33–1.22)	1.01 (0.39–2.62)	0.79 (0.32–1.94)
Non-Hispanic black	1.43 (1.02–2.02)	1.42 (0.78–2.56)	1.41 (0.97–2.05)	1.26 (0.64–2.48)	1.34 (0.45–4.01)	1.96 (0.55–7.02)
Other	0.93 (0.53–1.64)	1.13 (0.57–2.21)	0.73 (0.36–1.47)	0.70 (0.38–1.30)	1.62 (0.61–4.34)	3.39 (1.00–11.5)
Body mass index						
Normal	1.00	1.00	1.00	1.00	1.00	1.00
Overweight	1.82 (1.19–2.79)	1.44 (0.68–3.06)	1.65 (1.03–2.66)	1.43 (0.60–3.44)	2.78 (1.21–6.38)	1.58 (0.28–9.00)
Obesity	1.89 (1.30–2.74)	3.69 (2.09–6.52)	2.02 (1.35–3.04)	3.91 (1.99–7.70)	1.32 (0.55–3.15)	3.55 (1.13–11.2)
Poverty-to-income ratio						
<1.30	1.00	1.00	1.00	1.00	1.00	1.00
1.30–3.49	1.14 (0.81–1.60)	1.20 (0.77–1.87)	1.16 (0.79–1.69)	1.18 (0.72–1.92)	0.92 (0.44–1.92)	1.08 (0.38–3.01)
≥3.5	1.25 (0.84–1.84)	0.92 (0.48–1.77)	1.12 (0.73–1.73)	0.88 (0.42–1.86)	1.74 (0.76–4.00)	0.93 (0.33–2.64)
Dietary factors						
Energy, 100 kcal	1.02 (0.99–1.05)	1.02 (0.98–1.07)	1.03 (0.99–1.06)	1.05 (0.99–1.10)	0.97 (0.93–1.02)	0.93 (0.86–0.99)
Fat, % energy	1.01 (0.98–1.04)	1.01 (0.97–1.05)	1.01 (0.98–1.04)	1.02 (0.97–1.07)	0.99 (0.93–1.06)	0.95 (0.90–1.02)
Saturated fat, % energy	0.98 (0.93–1.03)	0.99 (0.91–1.08)	0.97 (0.92–1.03)	0.98 (0.88–1.08)	1.03 (0.92–1.15)	1.06 (0.96–1.17)
Fiber, g/d	0.98 (0.94–1.02)	1.04 (1.00–1.09)	0.98 (0.94–1.02)	1.04 (0.99–1.09)	0.97 (0.90–1.05)	1.04 (0.97–1.11)
Sodium, 100 mg/d	1.00 (0.99–1.01)	1.00 (0.97–1.02)	1.00 (0.99–1.02)	0.99 (0.96–1.02)	1.00 (0.98–1.03)	1.02 (0.97–1.06)
Potassium, 100 mg/d	1.00 (0.99–1.02)	0.95 (0.92–0.98)	1.00 (0.98–1.02)	0.94 (0.91–0.97)	1.03 (0.96–1.10)	1.01 (0.96–1.07)

Odds ratios (95% CI, confidence intervals) were calculated with the use of Logistic Regression. All covariables listed were included in the model simultaneously. All analyses adjusted for health insurance status.

### Factors associated with EBP elevated blood pressure and hypertension in US children and adolescents post COVID

Among US children aged 8 to 12 years old, those who were obese had higher odds of EBP than those with normal weight (OR, 3.21; 95% Cl, 1.63–6.33) (Table 3). A 100 mg/day increase in dietary sodium intake was also associated with higher odds of hypertension (OR, 1.05; 95% Cl, 1.01–1.10) (Table 3). No other factors such as age, gender, race, poverty-to-income ratio, or other dietary factors were significant. Among US adolescents aged 13 to 19, every additional year of age was associated with higher odds of both EBP (OR, 1.36; 95% Cl, 1.11–1.67) and hypertension (OR, 1.72; 95% Cl, 1.36–2.17) (Table 3). Additionally, girls had lower odds of EBP than boys (OR, 0.33; 95% Cl, 0.15–0.75). Adolescents who were overweight had higher odds of EBP (OR, 2.78; 95% Cl, 1.21–6.38) (Table 3). Those who were obese had higher odds of hypertension (OR, 3.55; 95% Cl, 1.13–11.2) (Table 3). No other factors such as race, poverty-to-income ratio, or dietary factors were significant.

## Discussion

In this nationally representative analysis using data from NHANES, the prevalence of both EBP and hypertension among U.S. youth declined from 1999 through 2011 in children (8–12 y) and adolescents (13–19 y), followed by a gradual increase thereafter. From the pre‑pandemic cycle (2015–2020) to the post‑pandemic cycle (2021–2023), we observed no increase in prevalence; in fact, hypertension decreased significantly among adolescents and showed a small decline among children over this interval. Sex differences were minimal in children, whereas dramatically among adolescents, with girls consistently exhibited approximately half the prevalence observed in boys. Differences by race/ethnicity were small in magnitude in both age groups.

Our results are consistent with prior NHANES‑based analyses reporting long‑term declines in pediatric EBP and hypertension through the late 2000s, with subsequent stabilization or modest increases thereafter. Notably, a 2021 analysis spanning 1999–2018 reported similar patterns, though comparability is limited because that study excluded children with BMI <5^th^ percentile and restricted the adolescent category to ≤17 years [19]. Trend comparisons across decades are complicated by the 2017 American Academy of Pediatrics (AAP) clinical practice guideline, which lowered diagnostic thresholds and excluded children with overweight/obesity from the normative tables. Applications of the new guideline to NHANES data consistently show higher prevalence relative to the 2004 Fourth Report and upward reclassification concentrated among youth with adverse cardiometabolic profiles [20]. Narrative reviews and methodological commentaries similarly conclude that switching to 2017 definitions increases the proportion classified with hypertensive BP, particularly among adolescents with obesity, making cross‑era comparisons sensitive to the chosen definition set [21]. Evidence around the COVID‑19 pandemic is mixed and depends on data source. Some clinical or EHR‑based studies reported reduced pediatric BP screening and follow‑up early in the pandemic and, in some settings, higher body weight and small BP increases. These factors could bias observed prevalence if measurement opportunities are fewer and risk is elevated in those who present [22].

The importance of maintaining healthy blood pressure trajectories in childhood is well established. Even in the absence of overt hypertension, higher pediatric systolic blood pressure is associated with adverse subclinical cardiovascular phenotypes in adulthood, including left ventricular hypertrophy [23], coronary artery calcification [24, 25], and subclinical atherosclerosis [26]. Evidence showed that brief bouts of moderate‑to‑vigorous physical activity can lower blood pressure in school‑age children, although these benefits may be attenuated by high sedentary time [27]. These data underscore the value of prevention and health promotion across settings where youth live, learn, and play.

Consistent with prior studies [1, 7], adiposity emerged as the most important risk factors for EBPEBP and hypertension in our study [28]. Given widespread concern about increases in pediatric obesity during and after the COVID‑19 period [29], one might have anticipated an accompanying rise in EBP and hypertension. Instead, we observed a trend of declined prevalence from 2015–2020 to 2021–2023, particularly among adolescents. Several explanations could account for this pattern, including secular changes in clinical practice or screening patterns, residual confounding, shifts in diet or physical activity that differentially affect blood pressure versus BMI, and methodological factors (e.g., participation patterns or measurement differences across cycles). These hypotheses warrant future investigations. In our exploratory analyses, a higher percentage of calories from fat was associated with greater odds of EBP among children (8–12 y). While adult studies often show weak or inconsistent associations between total fat intake and blood pressure [30], pediatric data remain limited. Given potential error in single‑ or short‑term dietary recalls and the heterogeneity of dietary fat sources, this finding should be interpreted cautiously and validated in analyses that distinguish fat subtypes and overall dietary quality.

### Strength and limitation

The strengths of our study include a large, nationally representative sample with blood pressures directly measured suing standardized protocols (i.e., 3 times per participants), age‑stratified analyses separating children from adolescents, and the inclusion of the most recent post‑pandemic cycle (2021–2023). We have also considered rich data on diet, physical activities and other covariates in the analysis. The evaluation of sex and race/ethnicity differences and consideration of behavioral and nutritional correlates add contextual value. Several limitations should be acknowledged. First, NHANES is repeated cross‑sectional study by design. Although we included large number of blood pressure related factors as covariates in this study (e.g., diet, physical activity), residual confounding cannot be completely ruled out. Second, we acknowledge the continuing decline in NHANES response rates and the possibility of residual bias. To minimize this, all estimates had employed cycle‑specific survey weighting and design variables; importantly, a previous nonresponse‑bias assessment did not detect major sources of bias [31]. In addition, race/ethnicity categories are heterogeneous; small subgroup sizes may have limited power to detect differences. Finally, differences in SBP and DBP measured by mercury sphygmomanometer and Omron 907XL in NHANES 2017–2018 and 2021–2023 required the addition of 1.5 mm Hg to oscillometric-measured SBP and the subtraction of 1.3 mm Hg from oscillometric-measured DBP to calibrate the values of the oscillometric to those of mercury devices [32, 33]. Regarding children and adolescents, the level of evidence-based validation still needs to be assessed to evaluate the suitability of these criteria in children and adolescents [34].

## Conclusions

We found that pediatric EBP and hypertension declined from pre-pandemic (2015–2020) to post- COVID-19 pandemic (2021–2023), particularly among adolescents. Sex differences were minimal in children but pronounced in adolescents, with lower prevalence among girls; race/ethnicity differences were small overall. Adiposity remains the dominant, modifiable correlation of abnormal blood pressure, reinforcing the need for integrated strategies that address healthy weight, routine blood pressure screening, physical activity promotion, and dietary quality in childhood and adolescence. Continued monitoring and methodologically rigorous studies are warranted to clarify post‑pandemic trajectories and to identify policy‑relevant levers that sustain improvements in pediatric cardiovascular health.

## Summary

### What is known about the topic

- Excess body weight is a strong, well-established correlate of elevated blood pressure (EBP) and hypertension in children and adolescents.
- COVID-19 disruptions were expected to worsen youth cardiometabolic risk via weight gain, reduced activity, and dietary shifts, but contemporary U.S. national data past 2020 have been limited.
- Higher sodium intake and poorer diet quality are linked to higher BP levels in pediatric populations.

### What this study adds

- In NHANES 1999–2023, EBP stayed stable or declined, and hypertension fell in both age groups.
- Adiposity remains the dominant modifiable correlate. From 2015-2023, obesity was strongly associated with higher odds of EBP/hypertension in children.
- In children, higher dietary fat and sodium were associated with higher odds; in adolescents, older age, male sex, and non-Hispanic Black race were additional risk factors, while EBP decreased numerically.

## Data Sharing Statement

All data are publicly accessible at https://www.cdc.gov/nchs/nhanes.
